# Student Engagement in Medical Research Curriculum Development Strategies: A Comprehensive Approach Utilizing Mixed-Methods Study and SWOT-TOWS-AHP Analysis

**DOI:** 10.1007/s40670-024-02226-2

**Published:** 2024-11-14

**Authors:** Sethapong Lertsakulbunlue, Panrawee Sertsuwankul, Kaophiphat Thammasoon, Kanlaya Jongcherdchootrakul, Boonsub Sakboonyarat, Anupong Kantiwong

**Affiliations:** 1 Department of Pharmacology, Phramongkutklao College of Medicine, Bangkok, 10400 Thailand; 2 Medical Student, Phramongkutklao College of Medicine, Bangkok, 10400 Thailand; 3 Department of Student Affairs, Phramongkutklao College of Medicine, Bangkok, 10400 Thailand; 4 Department of Military and Community Medicine, Phramongkutklao College of Medicine, Bangkok, 10400 Thailand; 5 Research Division, Armed Forces Research Institute of Medical Science, Royal Thai Army, Bangkok, Thailand

**Keywords:** Student engagement, SWOT, Analytic hierarchy process, Medical research, Curriculum development

## Abstract

**Introduction:**

Student engagement in medical curriculum development has become increasingly important, yet structured frameworks for decision-making and prioritizing strategies remain limited. This study demonstrates an approach integrating student representatives into strategy development to enhance engagement in medical research (MR) curriculum design and highlights students’ perspectives on MR.

**Methods:**

A mixed-methods study was conducted with 262 clinical year medical students and intern doctors. Participants completed a questionnaire on practices, perceptions, attitudes, motivations, and barriers related to MR. Focused group discussions evaluated beliefs towards MR and the faculty’s strengths, weaknesses, opportunities, and threats. Thematic analysis was performed. Strategies were developed using the TOWS matrix, and student representatives ranked the strategies using the analytic hierarchy process (AHP).

**Results:**

MR is perceived as complex and nonessential, yet its benefits in career development, patient care, and knowledge advancement are well-recognized. The chance to pursue research on personally interesting topics motivates student engagement in MR. However, curriculum overload has emerged as a significant barrier. The opportunity-strength strategy received the highest priority score of 0.33. The preferred sub-strategy involves establishing a centralized communication and coordination system to connect expert professors in both pre-clinical and clinical medical fields, both within and outside the institution, thereby enhancing the diversity of available mentors (priority score = 0.12).

**Conclusion:**

To address curriculum overload, a centralized communication system and coordination between students and experts are necessary. Additionally, cultivating time management skills is essential. Involving students in curriculum development ensures that their perspectives and needs are considered, leading to the creation of insightful strategies.

## Introduction

“Student Engagement in Schools” is a key aspect of the ASPIRE-to-Excellence initiative, initiated by the Association for Medical Education in Europe (AMEE) since 2012 to acknowledge and reward exceptional teaching practices [1]. Students could collaborate with faculty members across all academic domains, including research, teaching, governance, and curriculum development [2]. Moreover, actively incorporating student voices and involving student representatives in curriculum design and development offers valuable insights into student thinking, learning, and decision-making processes [3]. This input can guide the ongoing enhancement of teaching methods and materials, tailoring them to each medical school’s specific context [4].

Health-related research has consistently gained significance and has been widely promoted [5, 6]. However, there continues to be a global shortage of medical researchers despite the growing demand for them [7]. Over the past three decades, the proportion of medical researchers has sharply declined [8, 9]. Thus, the AMEE produced a guide titled “Developing Research Skills in Medical Students,” which recommends that every medical student understand research methods and the research benefits to address this challenge [9, 10]. This guide concluded that encouraging students’ active participation in research activities can greatly enhance their understanding of research [10].

Various studies have explored the attitudes, practices, knowledge levels, perceptions, motivations, and barriers related to research among medical and science students [11–18]. These factors are known to mediate engagement, as researchers are motivated to conduct studies based on their beliefs [19, 20]. This aligns with Self-Determination Theory, which posits that individuals are more likely to sustain behavior when driven by internal sources of motivation, such as their attitudes and perceptions, rather than external incentives [21]. Self-Determination Theory emphasizes fostering three core psychological needs—autonomy, competence, and a sense of belonging—for optimal motivation. Involving students in curriculum development for medical research (MR) enhances these needs, empowering them to take ownership of the process, build their skills, and deepen their engagement in MR activities [22, 23].

Previous frameworks often view students as customers or external stakeholders in university education rather than as team members [22]. Studies have shown that involving students in curriculum renewal teams and expanding their roles beyond traditional feedback—such as conducting literature reviews, writing reports, and proposing new courses—fosters their professional growth. This collaborative approach not only enhances students’ development but also creates a more inclusive and comprehensive curriculum design process [24, 25]. This is supported by previous research that successfully incorporated students as a primary stakeholder in program renewal [26], as well as medical curriculum development and evaluation [27]. Additionally, structured platforms for student engagement in curriculum evaluation and design enhance their awareness of academic medicine and interest in research careers [28]. Nevertheless, previous strategies and frameworks may lack detailed rankings of feedback strengths and weaknesses, making it challenging to prioritize new curriculum designs from the student’s perspective [22].

Identifying the curriculum’s strengths and weaknesses necessitates using an appropriate educational evaluation tool. Program evaluation frameworks have traditionally been categorized into systematic or naturalistic and decision-oriented or value-oriented approaches [29]. The Strengths, Weaknesses, Opportunities, and Threats (SWOT) analysis, known for its structured, systematic, and decision-oriented methodology, is remarkably adaptable for including student engagement in decision-making [30]. Furthermore, utilizing the TOWS matrix helps develop strategies to address weaknesses and threats while enhancing strengths and opportunities. This process aligns with Transformative Learning theory, enabling students to critically reflect on past learning challenges and contribute to meaningful curriculum development [31]. However, prioritizing the strategy derived from the SWOT analysis remains a challenge.

The analytic hierarchy process (AHP), initially introduced by Thomas L. Saaty for decision-making and ranking strategies with multiple options, has gained widespread recognition [32–35]. This approach has been effectively incorporated into the SWOT matrix across diverse industries, providing significant advantages in prioritizing strategies derived from SWOT analysis [34–36]. These tools have proven valuable in strategic planning for managers and executives, enhancing strategic decision-making [36, 37]. However, to our knowledge, the tool has been limitedly applied in the context of medical education. Thus, engaging students in both strategy development and the prioritization of newly developed strategies through the AHP could enhance their sense of belonging, competence, and autonomy, consistent with Self-Determination Theory [38]. This approach fosters long-term engagement with the strategies and enables students to gain deeper insights into the decision-making process. Moreover, by allowing students to monitor the implementation of these strategies, the co-creation process is further strengthened for future iterations [25, 38].

When developing curriculum strategies, it is crucial to incorporate key aspects such as student-centered, problem-based, integrated, clinically authentic, elective-driven, and systematic approaches in alignment with the SPICES model [39]. This study employed an explanatory mixed-methods design to explore the mediators of medical research engagement from the students’ perspective and enhance strategies for promoting research participation and publication among medical students. The objective was to examine the practices, attitudes, perceptions, barriers, and motivations of clinical year medical students and intern doctors who graduated from Phramongkutklao College of Medicine (PCM) toward conducting medical research. Furthermore, consistent with the SPICES model, the study aimed to leverage student-driven strategies to maximize medical research improvement policies’ effectiveness and clinical authenticity [4]. This was achieved through focus group interviews to assess the curriculum’s SWOT. Finally, the SWOT-TOWS-AHP analysis incorporated input from both medical students and intern doctors, facilitating problem-based critical reflection and yielding deeper insights. Given that PCM students enter directly from high school and may lack prior research experience, this systematic and structured framework is adaptable and well-suited to encourage and integrate student contributions effectively. The insights from this study could inform the development of a student-driven research curriculum framework and enhance the understanding of students’ mediators of research engagement within medical schools of similar contexts.

## Methods

### Study Design and Subjects

**Fig. 1:**
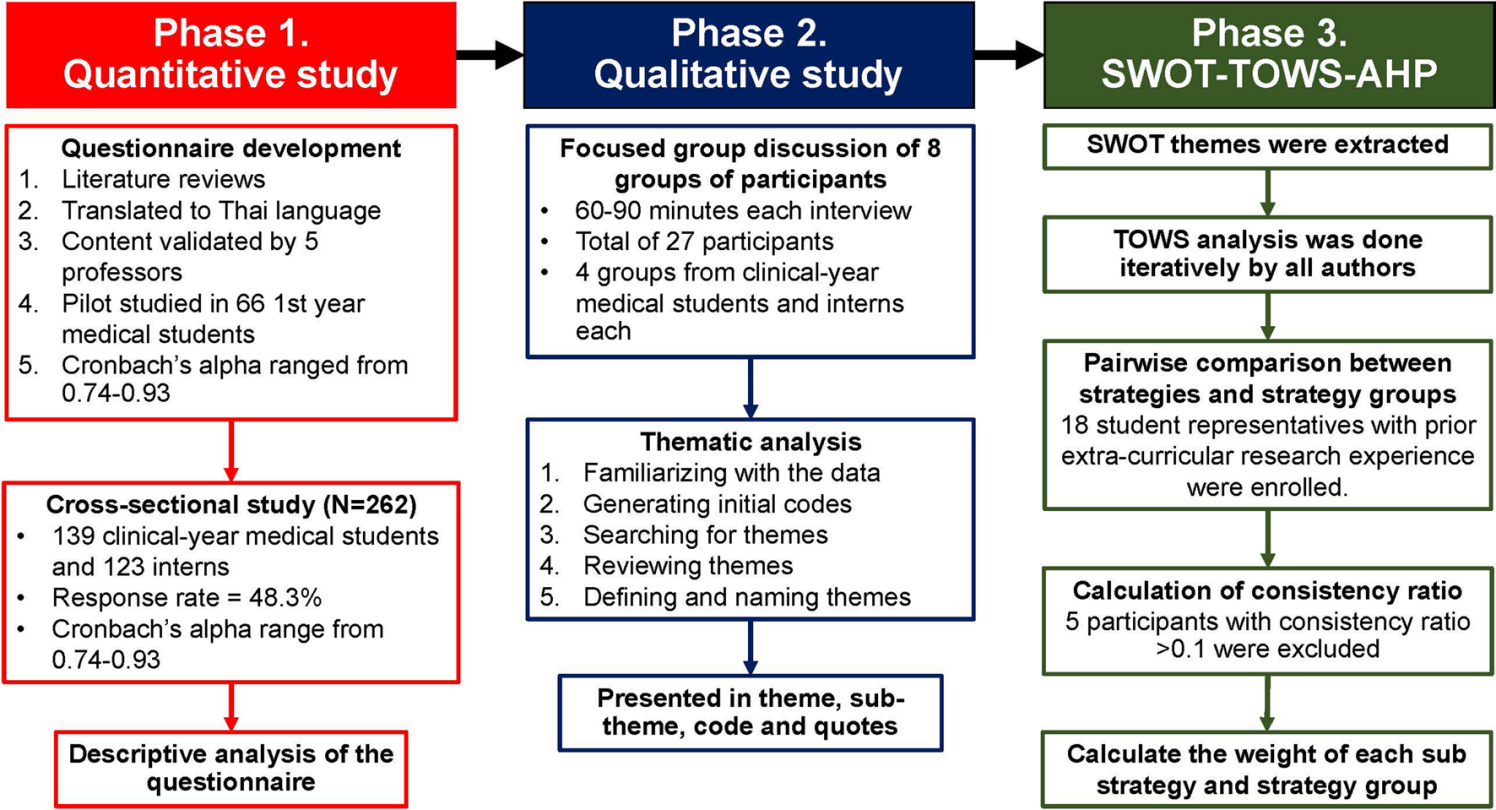
The flow of study process

The authors conducted an explanatory mixed-methods design and performed a SWOT-TOWS-AHP analysis to develop a student-driven strategy to improve further medical students’ involvement in medical research at PCM. The flow of this study is illustrated in Fig. 1.

### Quantitative Phase

A cross-sectional study was conducted at PCM, Bangkok, Thailand, using a self-administered survey. The survey was distributed to clinical year medical students, including fourth-, fifth-, and sixth-year students and intern doctors who had graduated from PCM. The total number of clinical year medical students was 292, with approximately 250 interns. Both groups were enrolled in the same curriculum paradigm and could present and publish their research findings voluntarily. Notably, many publications emerged from projects conducted during their fourth and sixth years as medical students and internships.

### Qualitative Phase

A focused group discussion (FGD) was conducted using purposive sampling to select clinical medical students and interns with previous extracurricular research experience. Eight groups, totaling 27 participants, were enrolled in the FGD, and semi-structured interviews were conducted. Each group consisted of 3–5 participants from the same year of education, with four groups of clinical year students and the remaining four groups of interns.

### SWOT-TOWS-AHP Analysis

Twenty-seven representatives were invited, and 18 (66.7%) representatives with prior MR experiences, including poster presentations, oral presentations, or MR publications, responded to the survey regarding pairwise comparison of the strategies. The student representatives included in the study were chosen based on their experiences as student year leaders, cadet commanders, MR group leaders, or presidents of academic or research clubs. These positions were determined through voting by the medical students, approved by the deans, and involved direct communication with the program director [40].

### Phramongkutklao College of Medicine Medical Research Curriculum

The PCM curriculum spans 6 years: 3 pre-clinical years dedicated to studying basic sciences and 3 clinical years focused on developing clinical experiences. In three specific years of the curriculum (the third, fourth, and sixth years of medical school), an introductory module on MR is mandatory.

In the third year, medical students learn the fundamentals of MR, including basic study designs focusing on quantitative methods, data analysis, and research proposal development. Additionally, students gain insights into aspects of public health, including community participation. Moving on to the fourth year, students delve deeper into advanced study designs and focus on conducting their own research study. Fourth-year students are divided into eight groups, each consisting of approximately 12, to develop and finalize a community-based research proposal, which is then presented as a report. Finally, in the sixth year, medical students are organized into pairs or groups of three to conduct medical research to improve medical care in a community hospital setting. Their research findings are presented, and a manuscript report is submitted.

### Data Collection

#### Quantitative Phase

We employed an electronic standardized questionnaire consisting of six parts—the first part comprised short-answer questions to collect demographic data. The rest consisted of a Likert scale questionnaire with five scores, addressing practices, perceptions, attitudes, barriers, and motivation toward MR. The questionnaire was based on relevant published works and the experience and context of PCM investigators. The detailed development, including the validity and reliability of the questionnaire, was published elsewhere [6].

Five expert professors reviewed the questionnaire to ensure content validity and reliability. Pilot testing was conducted among 66 first-year medical students, resulting in Cronbach’s alpha scores ranging from 0.74 to 0.93. Subsequently, the questionnaire was distributed to the study population. The final Cronbach’s alphas for practices, perceptions, attitudes, barriers, motivation, and overall questions were 0.83, 0.84, 0.74, 0.89, 0.88, and 0.93, respectively. Furthermore, the construct validity of the questionnaire revealed proper construct through confirmatory factor analysis [6].

#### Qualitative Phase

After analyzing the quantitative data, FGDs by SL and PS were conducted using a semi-structured interview format. Purposive sampling was employed to select clinical medical students and interns with prior extracurricular research experience, such as previous publications or international research presentations. This method enabled the collection of accurate, in-depth responses, as participants were able to provide comprehensive insights, ranging from attitudes toward research to the barriers they encountered [6]. The authors invited eligible participants to participate. The interviews included questions and probes to explore participants’ perceptions of MR, their previous experiences in MR, and the SWOT of PCM related to MR during their medical student years. Before conducting the qualitative research, interviewers received training at PCM. Informed consent was obtained from participants before the survey. Each interview lasted approximately 60 to 90 min and was conducted in Thai. Conversations were recorded and transcribed using the Zoom application. Before the analysis, two researchers (SL and AK) reviewed the transcriptions to identify any errors. The interviews were coded iteratively throughout the data collection process by SL, PS, and AK. Data collection continued until content saturation was achieved, indicated by the absence of new emerging codes in the final two groups of participants [41].

### SWOT-TOWS-AHP Analysis

A pairwise comparison survey was designed to evaluate each sub-strategy within the TOWS matrix and compare them among the four types of strategy groups: SO, WO, ST, and WT. The survey utilized a nine-point importance scale [42]. The authors explained the details of the survey, and it was administered through a self-answered online questionnaire.

### Statistical Analysis

#### Quantitative Phase

All data were downloaded from Google Forms and analyzed using *SPSS 29* (IBM, Armonk, NY, USA). Frequency distribution of demographic characteristics was performed. Categorical data were presented as percentages, and the Likert scale was presented as the median and interquartile range (IQR).

#### Qualitative Phase

A thematic analysis strategy focused on examining themes or patterns of meaning within data. This method can emphasize organization and detailed description of the dataset and theoretically informed interpretation of meaning [43]. The investigators (SL and AK) transcribed and proofread the interview recordings word for word. SL, PS, and KT followed a six-step guide for conducting thematic analysis [41]. First, we familiarized ourselves with the data and generated initial codes individually. Both inductive and deductive coding approaches were used, and the researchers discussed observed patterns and identified key themes. The themes of the students’ mediators toward conducting MR and SWOT were reviewed iteratively, and all authors assembled to define and name them. The final findings were presented as codes, subthemes, themes, and quotations.

### SWOT-TOWS-AHP Analysis

The combined strategies were formed by all authors using a TOWS matrix, incorporating the SWOT related to MR promotion in PCM, the students’ beliefs regarding conducting MR and a literature review. The AHP process consists of four steps. (1) Building the AHP hierarchical model to improve medical research participation and outcomes for medical students. (2) Conducting a pairwise comparison matrix to compare each sub-strategy and each strategic domain. The geometric mean consolidated the opinions since the participants' rankings carried equal weight [44]. (3) The consistency ratio (CR) calculation was performed, and participants with a CR over 0.1 were excluded. The CR is used to assess the consistency of judgments in the AHP, derived through the eigenvalue method for prioritization. It is calculated by dividing the consistency index (CI) by the random index (RI). The CI is defined as: “CI = (λ_max_ − *n*) / (*n* − 1)” where *λ*_max_ is the maximum eigenvalue and *n* is the size of the comparison matrix. A perfectly consistent matrix will have *λ*_max_ equal to *n*, with any deviation indicating inconsistency. The random index (RI) is a pre-calculated value representing the average consistency index of randomly generated pairwise comparison matrices based on the matrix size. For example, for size 3, 4, or 5 matrices, the RI values are 0.58, 0.90, and 1.12, respectively. The RI values increase as the matrix size grows, reflecting the increased likelihood of random inconsistency in larger matrices. The CR ensures that only judgments with an acceptable level of consistency (CR < 0.1) are used in the analysis [33]. (4) The overall weight of the components is calculated by multiplying the weight of each component within the component group.

## Results

### Quantitative Phase

Overall, 139 clinical year medical students and 123 interns participated in the study. The response rate was 47.6% and 49.2% for clinical year medical students and interns, respectively. 59.5% of the participants were male, and 22.1% had previously published an MR. The clinical year students and interns believe that MR enhances one’s career prospects, improves knowledge, and is valuable and complex, with a median Likert scale of four or higher. However, only the interns believe that MR promotes critical thinking and is essential for the medical profession, with a median Likert scale of four or higher.

Regarding the barriers, both groups hold the view that the lack of rewards or motivation and curriculum overload is the most problematic, with a median (IQR) Likert scale of 4 (3–4) and 4 (4–5), respectively. On the other hand, extrinsic motivations, such as the focus on pursuing higher degrees and the pursuit of further education, received a median Likert scale of 4 (3–4) (Table 1).

**Table 1:** Practices, perceptions, attitude, barriers, and motivation Likert-score stratified by educational level (*N* = 262)

Questions	Clinical year (*n* = 139)	Intern (*n* = 123)
	Median (IQR)	Median (IQR)
Practice		
Willingness to take part in any research related task	3 (3–3)	3 (3–4)
To spend more than 2 months on a research project	3 (2–3)	3 (2–3)
To devote the same time for medical research as their university studies	3 (3–4)	3 (2–3)
Perception		
Medical research promoting critical thinking	3 (3–4)	4 (3–4)
Enhancing one’s career prospect	4 (3–4)	4 (4–5)
Enhances knowledge	4 (3–4)	4 (3–4)
Research/publication should be mandatory	3 (2–3)	3 (2–4)
Research is important	3 (3–4)	4 (3–4)
Research experience should be a criterion for residency training	3 (2–3)	3 (2–3)
Attitude		
Medical research is valuable	4 (3–4)	4 (3–4)
Medical research is exciting	3 (2–3)	3 (2–3)
Medical research is enjoyable	3 (2–3)	3 (2–3)
Medical research is complicated	4 (3–5)	4 (3–5)
Medical research is time consuming	3 (3–4)	3 (2–3)
Medical research is essential for medical profession	3 (3–4)	4 (3–4)
Barrier		
Lack of allotted time	3 (3–4)	3 (3–4)
Lack of exposure and opportunities	3 (3–4)	3 (3–4)
Lack of training and support	3 (3–4)	3 (3–3)
Lack of mentoring and guidance	3 (2–4)	3 (2–3)
Lack of funding	3 (3–4)	3 (2–4)
Lack of personal knowledge of research process	3 (3–4)	3 (3–4)
Lack of statistical support	3 (3–4)	3 (3–4)
Lack of rewards or motivations	4 (3–5)	4 (3–4)
Curriculum overload	4 (3–5)	4 (3–5)
Motivation		
Focus on pursuing higher degrees	4 (3–4)	4 (3–4)
Formal recognition by university	4 (3–4)	3 (3–4)
Pursuit of further education	4 (3–4)	4 (3–4)
Pursuit of personal interest	3 (2–3)	3 (2–4)
Improving their potential in research skill	3 (2–4)	3 (3–4)
Having mentor guidance/role model	3 (2–4)	3 (3–4)
To be a part of help in solving medical problems in society	3 (2–4)	3 (3–4)

*QR* interquartile range

### Qualitative Phase

Table 2 demonstrates the theme, sub-theme, and codes of the thematic analysis of eight focus groups (FGD01 to FGD04 are groups consisting of clinical year students; FGD05 to FGD08 are interns).

**Table 2:** Thematic analysis of focused group discussion involving 8 groups of clinical year medical students and interns

Theme	Sub-theme	Code	*N*^a^
Perceptions and attitudes	View medical research as unimportant for medical student	Distant matter; do not need to engage in research; no desire to seek new knowledge beyond clinical practice; prefer patient care; further education tends to prioritize grades over research work; burdensome	8
	Medical research involves complexity and requires significant effort	Overly complex for medical students; time consuming; reading research articles is challenging; tired; requires a specialist level of expertise; high level of ability is required	8
	Research has benefits in terms of patient care and advancing knowledge	Beneficial for patients; necessary; generating new knowledge; doctors should engage in research to update their knowledge	4
	Research helps develop skills and is something that doctors should be knowledgeable about	Development of knowledge; enhance skills in various domains; basic statistical analysis is important; evidence-based medicine is important; research process knowledge is mandatory; research experience is mandatory	4
	Happiness in the process of conducting research	Enjoyable feeling during research discussions; joy when significant research findings emerge; enjoyment in data collection and statistical analysis	3
Barrier	Lacking skills in initiating and conducting research	Lacking confidence; initiating research topics independently is challenging; limited clinical experience; lack statistical analysis skills; lack skills to gather secondary data; limited proficiency in essential software tools; lack English language proficiency	8
	High workload and numerous responsibilities to fulfill	Too many extracurricular activities; too much workload throughout the day; too much homework; difficult time management	8
	Not being the owner of one's own research or being compelled to conduct it	Not feel a sense of ownership over the research work; not conducting research on a topic of personal interest; compelled to conduct research	7
	The lengthy and complex documentation process drains one's energy	Research ethics necessitate extensive documentation; document-related tasks are time-consuming and complex; delayed ethical approvals impede progress	6
	Lack of knowledge in accessing organizational resources and personnel	Unaware of professors who share similar interests; unaware of the capabilities of faculty laboratory equipment; unaware of the diverse research practices within the faculty	5
	Fear of disappointment and pressure from mentors, co-researchers or team members	Fear of criticism and rejection; fear of failure; fear of pressure and judgment from mentors; seniority culture; lack of collaborative teamwork; coordinating research with many individuals causes stress	5
	Lack of financial resources hinders research endeavors	Lack of financial resources; expensive open access publication fees; funding retrieval system access difficulty	2
Motivation	Having a close and consistent mentor/role model who constantly supports and nurtures	Good mentor/role model; examples from peers; senior/alumni as advisors; mentor with a flexible schedule; encouraging mentor; approachable and friendly mentors; mentor who motivates to engage in research	8
	Having the opportunity to conduct research on a topic of personal interest and being the project initiator	Sense of ownership; personal interest and curiosity; recognizing the significance of the engaged research work; to help a real-life case; newly founded gap of knowledge	7
	To use as a supplement for further education and professional advancement	Help establish valuable connections; research profile for further academic pursuits; professional advancement; academic ranks; enhance one's application for further education	6
	To enhance skills and expand one's knowledge	To learn the research process; acquire new knowledge; develop one's own skills; challenge oneself in research endeavors; deepen specialized knowledge	5
	Having a positive research experience in the past	Prior experience as a research collaborator; non-stressful working environment; sense of accomplishment and pride; success in presenting or publishing	5
	Participating in the academic presentation and building connections	Participating in academic conferences; opportunities and allows for networking with like-minded individuals; present research internationally	3

^a^The number of groups that provided responses related to the subtheme

#### Perceptions and Attitudes

Although some participants enjoyed conducting medical research, a prevalent belief among all groups was that MR is unnecessary for medical students, describing it as a complex and demanding task. A participant articulated, “It’s like a top-level subject, complex even after trying to learn it” (FGD02). Many argued that existing treatment guidelines suffice, negating the need for new research: “Traditional methods cure diseases; there's no need to create new knowledge” (FGD07). However, several acknowledged MR’s role in enhancing patient care and advancing medical knowledge, asserting doctors’ importance in being well-versed in research fundamentals: “Clinic practices and treatments are all based on research” (FGD04).

#### Barriers

Participants cited significant barriers, including high workloads that deter research activities, especially near exams: “If it’s close to the exam…I won’t do any research” (FGD08). They also expressed a lack of necessary skills and resources to initiate research, with one noting the challenge of tackling advanced topics like drug resistance (FGD06). The absence of personal research ownership and insufficient understanding of research topics led by instructors were further highlighted as impediments.

#### Motivations

Despite mentors receiving low ratings in the quantitative analysis, those with prior research experience stressed the value of having a supportive and consistent mentor, particularly in overcoming complex challenges: “The professor helps us manage complex issues” (FGD08). The autonomy to explore personally interesting topics was a key motivator, with participants feeling more passionate and engaged: “Pursuing personal interests leads to research driven by enthusiasm” (FGD04). Curiosity about knowledge gaps in existing guidelines also spurred interest: “Exploring guidelines continuously reveals gaps” (FGD07).

Furthermore, participants recognized MR as beneficial for further education and professional development, viewing it as a profile enhancer demonstrating expertise and genuine interest in a specialized field: “It serves as supporting evidence for further studies or specialization” (FGD01). Collaborative research with teachers was seen as a way to deepen knowledge and strengthen faculty relationships.

### SWOT-TOWS-AHP Analysis

**Fig. 2:**
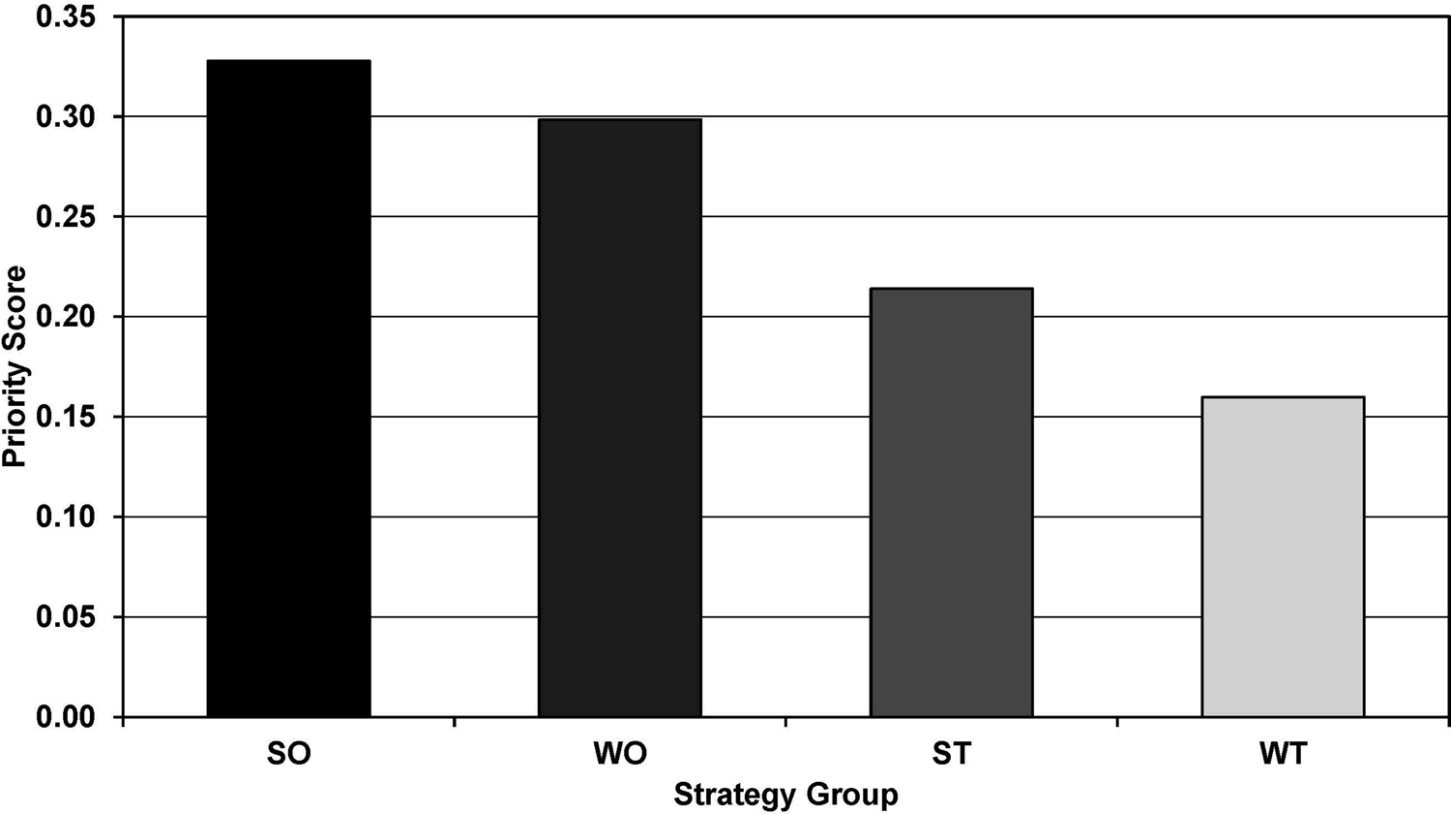
Priority score of each strategy group in the TOWS matrix: SO, Opportunity-Strength; WO, Opportunity-Weakness; ST, Threat-Strength; WT, Threat-Weakness

**Fig. 3:**
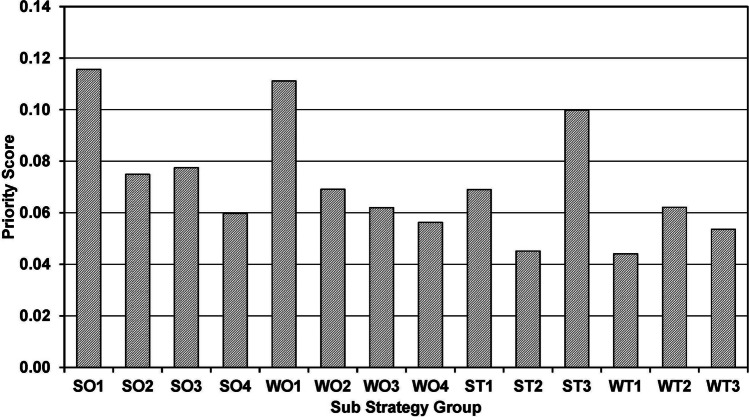
Priority score of each sub-strategy in the TOWS matrix: SO, Opportunity-Strength; WO, Opportunity-Weakness; ST, Threat-Strength; WT, Threat-Weakness. The codes for the sub-strategy groups are detailed as the strategy group followed by the strategy number. For example, “SO1” refers to sub-strategy number 1 from the Opportunity-Strength group

The SWOT-TOWS matrix containing the strategies derived from the SWOT analysis, mixed-methods studies, and literature review is presented in Table 3. For the AHP analysis, 18 participants with prior experience in research and a student representative (10 clinical year students and 8 interns) successfully engaged in pairwise comparisons of the strategies to determine the most ideal one. Five participants with a CR above 0.1 were excluded. Table 4 displays that the Opportunity-Strength (SO) strategy group is the most important, with a priority score of 0.33. Figure 2 illustrates the priorities of each strategy group. The sub-strategy “Developing a centralized communication and coordination system that connects expert professors in pre-clinical and clinical medical fields, both within and outside the institution” (SO1) is the most preferred, followed by the “Develop a faculty database system that provides information on their respective areas of expertise, the needs of medical students for research collaboration in various topics, the potential of resources, and funding sources available to support research work” (WO1) strategy. The priority scores for each sub-strategy are shown in Fig. 3.

**Table 3:** SWOT-TOWS analysis

SWOT analysis	Strengths (S)	Weaknesses (W)
	*What do you do well? What resources do you have within your control?*	*What needs to be enhanced to increase program success? What intrinsic factors place your program at a disadvantage?*
	S1. Teachers are knowledgeable, kind-hearted, and ready to provide guidance	W1. Lack of promotion regarding the research potential of both faculty and resources
	S2. Organizations and resources provide strong support for research endeavors	W2. Lack of collaboration from various disciplines in conducting research among medical students
	S3. High potential in the field of community medicine	W3. The research curriculum lacks flexibility and requires excessive time for course management
	S4. A rigorous and high-quality research and statistical analysis curriculum	W4. The funding system for medical students is difficult to access
	S5. Frequent conferences for presenting diverse research studies	W5. Not aware of the potential of community hospitals for conducting research in the curriculum
Oppurtunities (O)	Opportunity-Strength Strategy (SO)	Opportunity-Weakness Strategy (WO)
*What external factors could benefit your program?*	*How can strengths be used to take advantage of opportunities?*	*How can you minimize/overcome weaknesses by using opportunities?*
O1. Professors with diverse expertise who have not yet promoted research among medical students	SO1. Developing a centralized communication and coordination system that connects expert professors in pre-clinical and clinical medical fields, both within and outside the institution (S1/S2/O1/O2/O3)	WO1. Develop a faculty database system that provides information on their respective areas of expertise, the needs of medical students for research collaboration in various topics, the potential of resources and funding sources available to support research work (W1/W2/O1/O2/O3)
O2. Resident and fellow physicians who engage in research work and continue to advance	SO2. Support collaborative research in community medicine between medical students, resident and fellow physicians, and external individuals from diverse fields, fostering cooperation and knowledge exchange (S3/S4/O1/O2/O3)	WO2. Simplify the expense reimbursement system for medical students conducting research, both within and outside the curriculum, with the support of faculty advisors, resident or fellow physicians as mentors (O1/O2/W1/W2/W4)
O3. Connections with both local and international universities	SO3. Expand the scope of research to encompass a broader range of population groups, such as military hospitals or associate hospitals in various other areas, to include a larger and more diverse population (S3/S4/O4)	WO3. Allow for the utilization of research from other disciplines beyond the coursework-specific research for the purpose of credit transfer and evaluation in relevant subjects (O1/O2/O3/O4/W1/W2/W3/W5)
O4. Databases and accessibility on diverse population groups	SO4. Organize conferences to present collaborative research findings conducted jointly by medical students and resident physicians or fellow physicians (S2/S5/O2)	WO4. Establish a Memorandum of Understanding (MOU) for collaborative research between medical students and institutions, promoting data collection and collaboration to obtain representative samples on a larger scale (O3/O4/W1/W2/W4)
Threats (T)	Threat-Strength Strategy (ST)	Threat-Weakness Strategy (WT)
*What factors beyond your control place the program “at risk?”*	*How can you use program strengths to minimize threats?*	*How can you use threats to minimize weaknesses and use weaknesses to avoid threats?*
T1. Requesting research ethical use of extensive, lengthy, and complex documents	ST1. Develop a centralized system for documenting and addressing common ethical issues encountered in research, providing guidelines and solutions based on the experiences of experienced faculty members or senior peers (T1/T2/T3/S1/S2)	WT1. Enhance flexibility in medical student research within the curriculum by allowing the use of secondary data, reducing complexities in ethical considerations, and addressing time and budget constraints (T1/T2/W3/W4)
T2. The workload in the medical graduate program is substantial	ST2. Develop an extended mentoring system for encouraging medical students to engage in research (S1/S4/T1/T3)	WT2. Support research continuity and expansion by leveraging previous research proposals and addressing research issues while simplifying the ethical approval process and reducing the workload (T1/T2/T3/W1/W2/W4/W5)
T3. Seniority culture	ST3. Promote medical student research presentations at conferences and encourage publication in national and international journals while fostering innovation and creativity (S1/S2/S5/T1/T2)	WT3. Reduce curriculum workload to allow for more time to study important research issues and areas of interest in advance at community hospitals while assessing research capabilities of personnel and necessary resources (T1/T2/W3/W5)

The SWOT-TOWS matrix is adapted from Topor et al.

**Table 4:** Priority of each strategy and sub strategy in TOWS matrix by analytic hierarchy process analysis

Strategy group	Strategy group's priority	Sub strategy	Sub strategy's priority	Overall priority	Ranking of strategy
SO	0.33	SO1	0.35	0.1156	1
		SO2	0.23	0.0749	5
		SO3	0.24	0.0775	4
		SO4	0.18	0.0597	10
WO	0.30	WO1	0.37	0.1111	2
		WO2	0.23	0.0691	6
		WO3	0.21	0.0620	9
		WO4	0.19	0.0563	11
ST	0.21	ST1	0.32	0.0690	7
		ST2	0.21	0.0452	13
		ST3	0.47	0.0998	3
WT	0.16	WT1	0.28	0.0441	14
		WT2	0.39	0.0621	8
		WT3	0.34	0.0536	12

*SO* Opportunity-Strength, *WO* Opportunity-Weakness, *ST* Threat-Strength, *WT* Threat-Weakness

## Discussion

This study successfully employed a mixed-methods approach to determine the mediators of research engagement among medical students and interns. Although previous studies have provided strategies to foster faculty research for students [45–48], the present study stands out for its comprehensive design, which utilizes SWOT-TOWS-AHP analysis to integrate student engagement and develop and prioritize strategies. Students with previous experience in medical research were requested to provide their perspectives on the faculty’s strengths, weaknesses, opportunities, and threats. Additionally, student representatives were formally invited to rank the strategies formulated through the SWOT-TOWS analysis. Implementing a well-defined framework that delineates students’ roles and responsibilities could enhance their engagement in shaping the educational curriculum [49]. Thus, this study demonstrates the involvement of student representatives as co-creators in curriculum design, which offers the advantage of direct interaction with students, allowing for a deeper understanding of their thoughts and viewpoints [2, 3].

Various strategies have been implemented to enhance medical research engagement, including electives, mandatory courses, and summer research programs [50]. Similar to the student’s expectations in the present study, a pilot research training network for undergraduate students was established in 2015. This network facilitated collaboration between medical students, junior trainees, and senior staff members in medical education research [51]. Establishing a robust collaboration network to enhance staff accessibility would significantly benefit student engagement, as a major barrier to engagement is the perceived inaccessibility of staff [52].

Comprehensive reviews of effective strategies for enhancing research capacity have recommended various approaches [47]. These include recruiting new faculty research assistants, implementing mentoring programs, supporting local mentors, expanding and diversifying the mentor pool, actively engaging faculty mentors as stakeholders, and establishing a campus-wide resource [46, 47]. However, despite mentors’ significant role in these strategies, a systematic framework to fully leverage their potential remains unfulfilled [53]. Hence, it is strongly recommended that a well-structured system be established to drive the effective implementation of these strategies.

Regarding perception, previous literature from various countries has shown that research is often perceived as important and mandatory among undergraduate and postgraduate students and teaching staff [12, 15, 54]. Consistent with these findings, the current study also uncovered that participants perceive medical research as carrying multiple benefits. However, some participants may perceive medical research as overly complex, time-consuming, and unnecessary for all medical students. Additionally, certain individuals may prioritize dedicating their time to learning and memorizing practice guidelines, possibly due to a lack of awareness among many medical students regarding the importance of research in healthcare [12, 50]. Consequently, providing medical students with opportunities to conduct medical research is crucial.

Published research frequently identifies the shortage of time as a significant obstacle to conducting medical research, resulting in a decline in the number of medical students interested in research endeavors. However, it is imperative to acknowledge that time is an uncontrollable constraint, necessitating the provision of time management strategies for undergraduate researchers. For instance, implementing techniques such as establishing attainable objectives, prioritizing tasks based on their significance, and refining the planning process can assist in maintaining focus on a research project [55]. Additionally, involving a team, employing creative problem-solving strategies to overcome obstacles, and proactively managing potential distractions can aid in sustaining concentration [56]. Moreover, to address this barrier, speeding up medical research undertaken by students, as exemplified by the WT2 strategy, “Support research continuity and expansion by leveraging previous research proposals and addressing research issues, while simplifying the ethical approval process and reducing workload,” may prove beneficial.

In the present study, extrinsic motivation is the main driving force behind participants' engagement in medical research. Extrinsic motivations originate externally through incentives, bonuses, promotions, and rewards [57]. Similar studies have also yielded comparable findings, highlighting that extrinsic motivation is significant in motivating researchers, including career advancement, pursuit of further education, and recognition [58, 59]. However, according to the Self-Determination Theory, focusing excessively on extrinsic motivation can potentially hamper performance by negatively impacting intrinsic motivation [60].

Three fundamental needs must be fulfilled to empower motivation and encourage students’ engagement in medical research: autonomy, competence, and a sense of belonging [60]. Faculty members can support these needs by offering extracurricular research time, allowing students to choose their research topics and mentors, and, when feasible, hiring students as research assistants to enhance their autonomy [21]. To foster competence, it is beneficial to introduce research skills early on and provide practical training [21]. Additionally, participants in this study expressed that the opportunity to conduct research on a personally interesting topic and take the initiative in a project greatly motivates them to engage in medical research, thus promoting their sense of belonging.

Unlike Western medical programs, Thailand’s 6-year medical curriculum allows direct entry from high school, often leaving students with limited research experience [61, 62]. While the Medical Research Curriculum at PCM has led to successful student publications and international presentations, it is perceived as contributing to curriculum overload [6]. This study highlights that students may prioritize grades over research due to the heavy curriculum and lack of experience. To alleviate these burdens, barriers to research engagement must be identified and addressed, tailored to each institution [22]. Aligning with the SPICES model, medical research engagement should be student-centered, elective-driven, and systematic [39]. The newly developed strategies aim to foster a supportive ecosystem for medical research, integrating diverse topics, interprofessional collaboration, extracurricular activities, and centralized systems that connect students with experts, offering guidance on ethical proposal writing, especially for those new to research.

The present study has encountered several limitations. Firstly, the study focused exclusively on medical students and interns from PCM, which may limit the generalizability of the findings to other universities with different curricula. Therefore, differences in the ranking of strategies may occur, and external validation is needed. Secondly, since participation in the study was voluntary, selection bias could be a significant limitation. Only those who chose to participate were included, potentially influencing the results. Furthermore, the participants primarily consisted of experienced students, which may limit the applicability of these strategies to less skilled individuals. However, these strategies should promote further engagement in medical research and create valuable opportunities. Thirdly, although student engagement is emphasized, the study does not explicitly mention the involvement of other key stakeholders, such as faculty members or administrative staff, in developing the strategies. Broader stakeholder involvement would ensure the strategy addresses student needs and the institutional capacity to implement them effectively. Collaboration with faculty and administrative representatives could enhance the feasibility and sustainability of the proposed strategies. Lastly, when ranking the TOWS strategy, only a limited number of participants were involved, which may not fully represent the entire population. However, it is worth noting that selecting experienced and knowledgeable judges is more crucial than having many judges. Their expertise ensures accurate judgments without diluting the quality by involving individuals who may be less appropriate [63]. In the current study, student representatives with prior experience in medical research were selected, leading to more comprehensive opinions on ranking medical research strategies.

Despite these limitations, the present study also has notable strengths. It outlines a comprehensive framework for student engagement in the design of medical research curricula and identifies the support that faculty should provide. Furthermore, the study employs a comprehensive approach to understanding participants’ perceptions, barriers, and motivations toward medical research.

## Conclusion

The research utilized a mixed-methods study to demonstrate medical students’ and interns’ perceptions, attitudes, barriers, and motivations toward medical research. While medical research is considered complex and unnecessary for all students, it is also recognized as beneficial for enhancing career paths, improving patient care, and advancing knowledge. The opportunity to research a personally interesting topic motivates the participants to engage in medical research, but lacking the skills to initiate their own research is a significant barrier. Therefore, gradual and close support from mentors is needed. Moreover, the SWOT-TOWS-AHP analysis provided a strategic framework to prioritize and develop strategies for student engagement in medical research. The study offers valuable insights and a comprehensive framework for integrating student representatives into the design of medical research curricula.

## Data Availability

The datasets used and/or analyzed during the current study available from the author on reasonable request (contact Sethapong Lertsakulbunlue via Sethapong.ler@pcm.ac.th).

## Abbreviations

MR: Medical research
IQR: Interquartile range
PCM: Phramongkutklao College of Medicine
AHP: Analytic hierarchy process

## References

[1] Patricio M. The ASPIRE initiative: excellence in student engagement in the school. Educación Médica. 2016;17:109–14.

[2] Milles LS, Hitzblech T, Drees S, Wurl W, Arends P, Peters H. Student engagement in medical education: a mixed-method study on medical students as module co-directors in curriculum development. Med Teach. 2019;41:1143–50. 31203695 10.1080/0142159X.2019.1623385

[3] Anderson I. Student representation in managing the medical curriculum. Clin Teach. 2006;3:154–7.

[4] Peters H, Zdravkovic M, João Costa M, Celenza A, Ghias K, Klamen D, et al. Twelve tips for enhancing student engagement. Med Teach. 2019;41:632–7. 29683024 10.1080/0142159X.2018.1459530

[5] Sobczuk P, Dziedziak J, Bierezowicz N, Kiziak M, Znajdek Z, Puchalska L, et al. Are medical students interested in research? – Students’ attitudes towards research. Ann Med. 2022;54:1538–47. 35616902 10.1080/07853890.2022.2076900 PMC9891220

[6] Lertsakulbunlue S, Thammasoon K, Jongcherdchootrakul K, Sakboonyarat B, Kantiwong A. Practices, perceptions, attitudes, barriers and motivation and its impacts on research publication. Asia Pacific Scholar. 2023;8:23–5.

[7] Funston G, Piper RJ, Connell C, Foden P, Young AMH, O’Neill P. Medical student perceptions of research and research-orientated careers: an international questionnaire study. Med Teach. 2016;38:1041–8. 27008336 10.3109/0142159X.2016.1150981

[8] Davila JR. The physician-scientist: past trends and future directions. Mich J Med. 2016;1(1):66–73.

[9] Carberry C, McCombe G, Tobin H, Stokes D, Last J, Bury G, et al. Curriculum initiatives to enhance research skills acquisition by medical students: a scoping review. BMC Med Educ. 2021;21:312. 34078364 10.1186/s12909-021-02754-0 PMC8173745

[10] Laidlaw A, Aiton J, Struthers J, Guild S. Developing research skills in medical students: AMEE Guide No. 69. Med Teach. 2012;34:754–71. 10.3109/0142159X.2012.704438 22905661

[11] Memarpour M, Fard AP, Ghasemi R. Evaluation of attitude to, knowledge of and barriers toward research among medical science students. Asia Pac Fam Med. 2015;14:1. 25705121 10.1186/s12930-015-0019-2 PMC4336721

[12] AlGhamdi KM, Moussa NA, AlEssa DS, AlOthimeen N, Al-Saud AS. Perceptions, attitudes and practices toward research among senior medical students. Saudi Pharmaceut J. 2014;22:113–7. 10.1016/j.jsps.2013.02.006 PMC3950504 24648822

[13] Pallamparthy S, Basavareddy A. Knowledge, attitude, practice, and barriers toward research among medical students: a cross-sectional questionnaire-based survey. Perspect Clin Res. 2019;10:73. 31008073 10.4103/picr.PICR_1_18 PMC6463502

[14] El Achi D, Al Hakim L, Makki M, Mokaddem M, Khalil PA, Kaafarani BR, et al. Perception, attitude, practice and barriers towards medical research among undergraduate students. BMC Med Educ. 2020;20:195. 32552801 10.1186/s12909-020-02104-6 PMC7298799

[15] Faisal Fahim M. Perception towards research among undergraduate physical therapy students. Biom Biostat Int J. 2018;7(3):171–5.

[16] Osman T. Medical students’ perceptions towards research at a Sudanese University. BMC Med Educ. 2016;16:253. 27682259 10.1186/s12909-016-0776-0 PMC5041407

[17] Habineza H, Nsanzabaganwa C, Nyirimanzi N, Umuhoza C, Cartledge K, Conard C, et al. Perceived attitudes of the importance and barriers to research amongst Rwandan interns and pediatric residents – a cross-sectional study. BMC Med Educ. 2019;19:4. 30606184 10.1186/s12909-018-1425-6 PMC6318911

[18] Al-Shalawy FA-N, Haleem A. Knowledge, attitudes and perceived barriers towards scientific research among undergraduate health sciences students in the Central Province of Saudi Arabia. Educ Med J. 2015;7(1). 10.5959/eimj.v7i1.266.

[19] Lev EL, Kolassa J, Bakken LL. Faculty mentors’ and students’ perceptions of students’ research self-efficacy. Nurse Educ Today. 2010;30:169–74. 19682774 10.1016/j.nedt.2009.07.007 PMC2815262

[20] Kahu ER. Framing student engagement in higher education. Stud High Educ. 2013;38:758–73.

[21] Rosenkranz SK, Wang S, Hu W. Motivating medical students to do research: a mixed methods study using Self-Determination Theory. BMC Med Educ. 2015;15:95. 26032008 10.1186/s12909-015-0379-1 PMC4486085

[22] Kassab SE, Taylor D, Hamdy H. Student engagement in health professions education: AMEE Guide No. 152. Med Teach. 2023;45:949–65. 36306374 10.1080/0142159X.2022.2137018

[23] Wilson C, Sims S, Dyer J, Handley F. Identifying opportunities and gaps in current evaluation frameworks – the knowns and unknowns in determining effective student engagement activity. Assess Eval High Educ. 2022;47:843–56.

[24] Khan H, Smith EEA, Reusch RT. Shifting medical student involvement in curriculum design: from liaisons to cocreators. Acad Med. 2022;97(5):623–4. 10.1097/ACM.0000000000004622. 10.1097/ACM.0000000000004622 35476833

[25] Könings KD, Mordang S, Smeenk F, Stassen L, Ramani S. Learner involvement in the co-creation of teaching and learning: AMEE Guide No. 138. Med Teach. 2021;43:924–36. 10.1080/0142159X.2020.1838464 33153367

[26] Mirzazadeh A, Gandomkar R, Hejri SM, Hassanzadeh G, Koochak HE, Golestani A, et al. Undergraduate medical education programme renewal: a longitudinal context, input, process and product evaluation study. Perspect Med Educ. 2016;5:15–23. 26820748 10.1007/s40037-015-0243-3 PMC4754210

[27] Rooholamini A, Amini M, Bazrafkan L, Dehghani MR, Esmaeilzadeh Z, Nabeiei P, et al. Program evaluation of an Integrated Basic Science Medical Curriculum in Shiraz Medical School, using CIPP evaluation model. J Adv Med Educ Prof. 2017;5:148–54. 28761888 PMC5522906

[28] Geraghty JR, Young AN, Berkel TDM, Wallbruch E, Mann J, Park YS, et al. Empowering medical students as agents of curricular change: a value-added approach to student engagement in medical education. Perspect Med Educ. 2019;9:60–5. 10.1007/s40037-019-00547-2 PMC7012994 31823304

[29] Kapoor A, Mehta AH, Arobelidze S, Foshee CM. A Decision-oriented approach to evaluating a leadership curriculum in fellowship. ATS Sch. 2024;5:96–108. 38638919 10.34197/ats-scholar.2023-0003OC PMC11025398

[30] Antoniadou M, Kanellopoulou A. Educational approach: application of SWOT analysis for assessing entrepreneurial goals in senior dental students. Eur J Investig Health Psychol Educ. 2024;14:753–66. 10.3390/ejihpe14030049 PMC10969685 38534910

[31] Lockey A, Conaghan P, Bland A, Astin F. Educational theory and its application to advanced life support courses: a narrative review. Resusc Plus. 2021;5:100053. 34223327 10.1016/j.resplu.2020.100053 PMC8244342

[32] Saaty TL. What is the analytic hierarchy process? In: Mitra G, Greenberg HJ, Lootsma FA, Rijkaert MJ, Zimmermann HJ, editors. Mathematical models for decision support. NATO ASI Series. 1988;48:109–21.

[33] Saaty TL, Sodenkamp M. Making decisions in hierarchic and network systems. Int J App Decision Sci. 2008;1:24.

[34] Datta K. Application of SWOT-TOWS matrix and analytical hierarchy process (AHP) in the formulation of geoconservation and geotourism development strategies for Mama Bhagne Pahar: an important geomorphosite in West Bengal. India Geoheritage. 2020;12:45.

[35] Lee Y, Kim YJ, Lee MC. Improving public acceptance of H2 stations: SWOT-AHP analysis of South Korea. Int J Hydrogen Energy. 2021;46:17597–607.

[36] Benzaghta MA, Elwalda A, Mousa M, Erkan I, Rahman M. SWOT analysis applications: an integrative literature review. J Global Business Insights. 2021;6:55–73.

[37] Topor DR, Dickey C, Stonestreet L, Wendt J, Woolley A, Budson A. Interprofessional health care education at academic medical centers: using a SWOT analysis to develop and implement programming. MedEdPORTAL. 2018;14:10766. 10.15766/mep_2374-8265.10766 PMC6342375 30800966

[38] Ryan RM, Deci EL, editors. Self-determination theory: basic psychological needs in motivation, development, and wellness. Guilford Press; 2017;38(3):231.

[39] Harden RM, Sowden S, Dunn WR. Educational strategies in curriculum development: the SPICES model. Med Educ. 1984;18:284–97. 6738402 10.1111/j.1365-2923.1984.tb01024.x

[40] Meeuwissen SNE, Spruijt A, van Veen JW, de Goeij AFPM. Student participation in governance of medical and veterinary education: experiences and perspectives of student representatives and program directors. Adv Health Sci Educ. 2019;24:665–90. 10.1007/s10459-019-09890-9 PMC6775033 31044324

[41] Kiger ME, Varpio L. Thematic analysis of qualitative data: AMEE Guide No. 131. Med Teach. 2020;42:846–54. 10.1080/0142159X.2020.1755030 32356468

[42] Saaty TL. The analytic hierarchy process: decision making in complex environments. In: Avenhaus R, Huber RK, editors. Quantitative assessment in arms control. Boston, MA: Springer; 1984. 10.1007/978-1-4613-2805-6_12

[43] Braun V, Clarke V. Using thematic analysis in psychology. Qual Res Psychol. 2006;3:77–101.

[44] Aczél J, Saaty TL. Procedures for synthesizing ratio judgements. J Math Psychol. 1983;27:93–102.

[45] Golenko X, Pager S, Holden L. A thematic analysis of the role of the organisation in building allied health research capacity: a senior managers’ perspective. BMC Health Serv Res. 2012;12:276. 22920443 10.1186/1472-6963-12-276 PMC3464180

[46] Godreau IG-SJF-OMC-SJM; MVG-CJ. Growing faculty research for students’ success: best practices of a research institute at a minority-serving undergraduate institution. Journal of Research Administration. 2015;46:55–78

[47] Huenneke LF, Stearns DM, Martinez JD, Laurila K. Key strategies for building research capacity of university faculty members. Innov High Educ. 2017;42:421–35. 29225411 10.1007/s10755-017-9394-y PMC5722023

[48] Aithal PS. How to boost faculty research performance in HEI’s to improve intellectual property by integrating it with faculty compensation – a “theory of accountability” based framework. Int J Manag Technol Soc Sci. 2018;3(2):130–51.

[49] Hsih KW, Iscoe MS, Lupton JR, Mains TE, Nayar SK, Orlando MS, et al. The student curriculum review team: how we catalyze curricular changes through a student-centered approach. Med Teach. 2015;37(11):1008–12. 10.3109/0142159X.2014.990877 25532595

[50] Burgoyne LN, O’Flynn S, Boylan GB. Undergraduate medical research: the student perspective. Med Educ Online. 2010;15:5212. 10.3402/meo.v15i0.5212 PMC2939395 20844608

[51] Sideris M, Hanrahan J, Staikoglou N, Pantelidis P, Pidgeon C, Psychalakis N, et al. Optimizing engagement of undergraduate students in medical education research: the eMERG training network. Annals of Medicine and Surgery. 2018;31:6–10. 29922460 10.1016/j.amsu.2018.05.008 PMC6004769

[52] Deutschlander D. Enhancing engagement with faculty and staff to facilitate student success: an evaluation of a parent intervention. Educ Eval Policy Anal. 2019;41:239–59.

[53] Drake TM, Bath M, Claireaux H, Mohan M, Fitzgerald JEF, Dynes K, et al. Medical research and audit skills training for undergraduates: an international analysis and student-focused needs assessment. Postgrad Med J. 2018;94:37–42. 28866608 10.1136/postgradmedj-2017-135035

[54] Okoduwa SIR, Abe JO, Samuel BI, Chris AO, Oladimeji RA, Idowu OO, et al. Attitudes, perceptions, and barriers to research and publishing among research and teaching staff in a Nigerian research institute. Front Res Metr Anal. 2018;3(26):1–10.

[55] Claessens BJC, van Eerde W, Rutte CG, Roe RA. A review of the time management literature. Pers Rev. 2007;36:255–76.

[56] Chase J-AD, Topp R, Smith CE, Cohen MZ, Fahrenwald N, Zerwic JJ, et al. Time management strategies for research productivity. West J Nurs Res. 2013;35:155–76. 22868990 10.1177/0193945912451163

[57] Saini M, Kumar A, Kaur G. Research perception, motivation and attitude among undergraduate students: a factor analysis approach. Procedia Comput Sci. 2020;167:185–92.

[58] Ommering BWC, Wijnen-Meijer M, Dolmans DHJM, Dekker FW, van Blankenstein FM. Promoting positive perceptions of and motivation for research among undergraduate medical students to stimulate future research involvement: a grounded theory study. BMC Med Educ. 2020;20:204. 32586311 10.1186/s12909-020-02112-6 PMC7318757

[59] Ichsan I, Wahyuniati N, McKee R, Lobo L, Lancaster K, Redwood-Campbell L. Attitudes, barriers, and enablers towards conducting primary care research in Banda Aceh, Indonesia: a qualitative research study. Asia Pac Fam Med. 2018;17:8. 30065616 10.1186/s12930-018-0045-y PMC6064079

[60] Deci EL, Koestner R, Ryan RM. A meta-analytic review of experiments examining the effects of extrinsic rewards on intrinsic motivation. Psychol Bull. 1999;125:627–68. 10589297 10.1037/0033-2909.125.6.627

[61] Lam TP, Bess Lam YY. Medical Education Reform: The Asian Experience. Acad Med. 2009;84:1313–7. 19707080 10.1097/ACM.0b013e3181b18189

[62] Mei A, Gao D, Jiang J, Qiao T, Wang F, Li D. The medical education systems in China and Thailand: a comparative study. Health Sci Rep. 2022;5(6):e826. 10.1002/hsr2.826 PMC9529001 36210882

[63] Saaty TL, Özdemir MS. How many judges should there be in a group ? Annals of Data Science. 2014;1:359–68.

